# *Polygonatum odoratum* polysaccharide attenuates lipopolysaccharide‐induced lung injury in mice by regulating gut microbiota

**DOI:** 10.1002/fsn3.3622

**Published:** 2023-08-15

**Authors:** Jia‐rui Liu, Bo‐xue Chen, Mei‐ting Jiang, Tian‐yi Cui, Bin Lv, Zhi‐fei Fu, Xue Li, Yao‐dong Du, Jin‐he Guo, Xin‐qin Zhong, Ya‐dan Zou, Xin Zhao, Wen‐zhi Yang, Xiu‐mei Gao

**Affiliations:** ^1^ Key Laboratory of Pharmacology of Traditional Chinese Medical Formulae, Ministry of Education Tianjin University of Traditional Chinese Medicine Tianjin China; ^2^ State Key Laboratory of Component‐based Chinese Medicine Tianjin University of Traditional Chinese Medicine Tianjin China

**Keywords:** gut microbiota, immuno‐inflammation, lung injury, *Polygonatum odoratum* polysaccharide

## Abstract

*Polygonatum odoratum* is appreciated for its edible and medicinal benefits especially for lung protection. However, the contained active components have been understudied, and further research is required to fully exploit its potential application. We aimed to probe into the beneficial effects of *Polygonatum odoratum* polysaccharide (POP) in lipopolysaccharide‐induced lung inflammatory injury mice. POP treatment could ameliorate the survival rate, pulmonary function, lung pathological lesions, and immune inflammatory response. POP treatment could repair intestinal barrier, and modulate the composition of gut microbiota, especially reducing the abundance of *Klebsiella*, which were closely associated with the therapeutic effects of POP. Investigation of the underlying anti‐inflammatory mechanism showed that POP suppressed the generation of pro‐inflammatory molecules in lung by inhibiting iNOS^+^ M1 macrophages. Collectively, POP is a promising multi‐target microecological regulator to prevent and treat the immuno‐inflammation and lung injury by modulating gut microbiota.

## Graphical abstract

*Polygonatum odoratum* polysaccharide (POP) is a promising multi‐target microecological regulator to prevent and treat the immuno‐inflammation and lung injury by modulating gut microbiota.

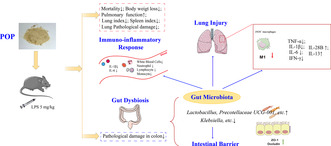

## 1 INTRODUCTION

*Polygonatum odoratum*, also known as fragrant Solomon's seal, is a species of flowering plant in the Asparagaceae family. In addition to the ornamental qualities, it is also highly valued for its edible and medicinal properties (Zhou et al., 2015). The rhizomes of the plant are consumed in a variety of dishes including the soups and stews in some cultures. This part of the plant is also used in traditional Chinese medicine (TCM) as Polygonati Odorati Rhizoma (Yu‐Zhu) with the efficacy in “nourishing yin and moistening dryness” and “producing saliva and quenching thirst,” which are commonly used in the treatment of lung diseases such as cold and dry cough and thirst due to diabetes. While the dried and powdered rhizomes can be used as a medicinal tea that is believed to have various health benefits, such as improving circulation, boosting immunity, and reducing inflammation (Committee for the Pharmacopoeia of PR China, 2020; Zhao et al., 2018). Despite its long history of being utilized both as the food source and herbal medicine with high nutritional value, the active components responsible for the efficacy of *P. odoratum* remain understudied. This constitutes a gap in the current understanding of this species and its potential application, warranting further research and investigation.

*Polygonatum odoratum* polysaccharide (POP), a main active component of *P. odoratum*, has been associated with antidiabetic, anti‐alcohol‐induced liver injury, and immunomodulatory effects (Liu et al., 2015; Zhao et al., 2019; Zhu et al., 2022). As biological macromolecules, most of polysaccharides cannot be directly absorbed by the gastrointestinal tract, but as a key carbon source required for the growth of probiotics, it can indirectly produce beneficial effects by promoting the proliferation of probiotics and converting them into active metabolites into the system (Zhang et al., 2022). POP has been reported to enhance species richness and improve the community structure of gut microbiota, characterized by reducing the relative abundances of *Clostridium*, *Enterococcus*, *Coprobacillus*, *Lactococcus*, and *Sutterella* in high‐fat diet‐induced obesity rats (Wang et al., 2018).

Inflammatory injury in lungs is initiated by a complex series of events. In some extreme cases, it can develop into the acute respiratory distress syndrome and multiple organ failure, threatening the patient's life. Even if the patients can survive, the pulmonary function has been seriously damaged and is difficult to be cured (Wang et al., 2020). Notably, alterations in the gut microbiota have been recognized in many immunoinflammatory disorders, and attenuation of the gut microbiota dysbiosis may have the positive effect on lung injury (Dhar & Mohanty, 2020). Although the host's immunoinflammatory response is a multi‐factor, multi‐step, and multi‐phase process, for the polysaccharides that is orally administrated in most cases, gut microbiota has been regarded as one of the key targets in the prevention and treatment of the immunoinflammatory disorders and the associated respiratory diseases (Li et al., 2021). However, the effects of POP on lung inflammatory injury via modulation of gut microbiota have not been investigated. We thereby presume POP might be a potential prebiotic to improve gut dysbiosis, which can lead to a reduction of systemic immune inflammation and contribute to the amelioration of lung injury.

In this work, we were aimed to investigate the effects of POP in alleviation of lung injury via gut microbiota modulation on lipopolysaccharide (LPS)‐induced inflammatory mice models. By employing 16S rRNA sequencing and fecal transplantation techniques, the relationship between gut microbiota composition and lung injury‐related parameters altered by POP was directly investigated. Furthermore, the anti‐inflammatory mechanisms of POP in lung associated with gut microbiota modulation were explored by multiplex cytokine analysis and immunofluorescent assays. Our findings may offer the biological basis for clarifying the “nourishing yin and moistening dryness” effects of Polygonati Odorati Rhizoma based on the gut, and contribute to the development of clinically available anti‐immunoinflammatory and lung protective agent for lung injury treatment.

## 2 MATERIALS AND METHODS

### 2.1 Chemicals and reagents

The material of *P. odoratum* (batch NO:191201091) was purchased from Hebei Chunkai Pharmaceutical Co., Ltd, China, and authenticated by Dr. Lijuan Zhang from Tianjin University of Traditional Chinese Medicine (Tianjin, China).

### 2.2 Preparation of *P. odoratum* polysaccharide

The POP was prepared by water extraction and the alcohol precipitation method according to previous study (Fu et al., 2020; Hu et al., 2022). Briefly, the material of *P. odoratum* was crushed into powder and refluxed twice with deionized water (liquid–solid ratio at 10:1) at 100°C for 2 h. The extracted solution (about 5 L) was pooled and further concentrated to 1/7 of the initial volume under reduced pressure at 50°C. The concentrated solution was combined and precipitated by adding 95% ethanol (to finial 80%, v/v), and standing at 4°C for 24 h. The resultant precipitate was lyophilized to obtain the total POP powder (with the extraction rate of 12.7%). The content of polysaccharides was determined by the sulfuric acid–phenol method (Fu et al., 2020), and POP was composed of 66.36% carbohydrates (Table S1).

### 2.3 Experimental animals

Male C57BL/6N mice (6‐week‐old, weighing 18–22 g) were purchased from Beijing Vital River Laboratory Animal Technology Co., Ltd (approval number: SCXK [Beijing] 2021‐0006). All mice were housed in the SPF environment (12 h light–dark cycle, 22 ± 2°C, and 40%–60% relative humidity) with the free access to water and food. All animal experiments strictly complied with the Guide for the Care and Use of Laboratory Animals and approved by the Animal Ethics Committee of Tianjin University of Traditional Chinese Medicine (TCM‐LAEC2021053).

### 2.4 LPS‐induced inflammatory model and POP administration

LPS injection induced lung injury in vivo due to a high immunoinflammatory response, and the lung injury models in this study were induced by reference to the previous study (Lv et al., 2023). After a week of adaption, the mice were randomly divided into six groups (15 mice for each): control (Con) group, LPS group, dexamethasone (DEX) group (5 mg/kg), and three levels of polysaccharides‐treated (POP‐100, ‐200, and ‐400 mg/kg) groups. Besides the Con group, all the others were injected with LPS into the tail vein at the dose of 5 mg/kg from the third to the fifth day of drug administration for modeling. The drugs of DEX and POP dissolved in double distilled water were given to mice once a day by gavage for 7 days. The Con and LPS group mice were received the same volume of double distilled water. All the mice were sacrificed under the deeply anesthetized condition after the morphology examinations and pulmonary function by the pulmonary system (software‐iox2, EMKA Technologies, France) (Zhang et al., 2021).

### 2.5 Fecal microbiota transplantation (FMT)

The FMT was performed under the anaerobic condition. Fresh feces from donors were pooled and homogenized, diluted in sterile saline with a final concentration of 100 mg feces/mL. The suspension was adequately mixed and placed stably for 10 min. Afterwards, the supernatant was collected and used for FMT. Before the FMT, the donors were treated with POP‐400 mg/kg (Con+POP400), and the receptors were treated with antibiotic cocktail of 0.5 g/L vancomycin, 1 g/L neomycin, 1 g/L metronidazole, and 1 g/L ampicillin for 3 days. Within the next 7 days, FMT group receipted intragastric administration of the supernatant from donor mice (10 μL/g) once a day.

### 2.6 Immunoinflammatory assays

The numbers of the immune cells, including the white blood cell (WBC), lymphocyte (LY), neutrophil (NE), and monocyte (MO), were tested immediately by an automated hematology analyzer (MEK‐7222K) in the blood of mice, which was collected before the euthanasia using the eyeball‐extraction method and incubated with EDTA at the room temperature. The cytokine concentrations of TNF‐α, IL‐6, and IL‐1β in serum and lung tissue, were determined by the corresponding ELISA kits (Sinobestbio), and detected by the spectrophotometry (UV‐3100, Mapada).

### 2.7 Tissue pathological examination

Lung, spleen, and colon tissues of mice were collected, washed with PBS, drained well with the filter paper, and then weighed. The relative weight of tissue was equal to the weight of the tissue divided by the body weight. The tissues were snap‐frozen in liquid nitrogen for further analysis. Lung and parts of the colon were fixed in 4% paraformaldehyde for 48 h, embedded in paraffin and cut into 5‐μm slices, and stained with hematoxylin–eosin (H&E). The pathological injury of lung and colon tissues was observed and imaged by the light microscope (Leica DM750).

### 2.8 Immunofluorescent assays

The colonic and lung segments were cut out and fixed with phosphate‐buffered formalin and embedded in paraffin. Briefly, paraffin‐embedded sections of colon and lung tissue were cut into 5 μm thickness. Immunofluorescent staining was performed according to the standard instructions (Xie et al., 2021). The slices were rewarmed at 60°C for 60 min before the experiment, and then subjected to dewaxing and hydration. Before staining, sections were permeabilized with PBS containing 3% Triton X‐100 at room temperature for 30 min and heated for 10 min in EDTA buffer to repair antigen. Nonspecific binding sites were blocked by incubation with 5% blocking goat serum in washing buffer at 37°C for 30 min. Afterwards, the sections were incubated with primary antibody at 4°C for overnight, and then exposed to secondary antibody conjugated with the appropriate fluorescein at room temperature for 30 min. The primary antibodies and dilution ratios were listed as follows: zonula occludens‐1 (ZO‐1, 1:200, Abclonal, Cat.NO: A0659), occludin (1:100, Abclonal, Cat.NO: A2601), iNOS (1:100, Proteintech, Cat.NO: 80517‐1‐RR), and CD206 (1:400, Servicebio, Cat.NO: GTX42264). The secondary antibodies and dilution ratios were listed as follows: goat anti‐rabbit‐Alexa Fluor 594 (1:200, BOSTER, BA1142), goat anti‐rabbit‐Alexa Fluor IgG (1:200, Abcam, ab150077), and Fluorescein‐conjugated affinipure goat anti‐rat IgG (1:100, Proteintech, SA00003‐11). After staining with DAPI for 10 min, the pathological sections were photographed with a light microscope (Pannoramic MIDI, 3DHISTECH Company Limited) with a case viewer imaging system.

### 2.9 Microbial diversity analysis

Fresh fecal samples were collected before the administration, modeling, and euthanasia under sterile condition, and immediately frozen at −80°C. The microbial genome DNA was extracted, and the 16S rRNA genes of V3–4 regions were amplified using primers 338F and 806R, and then purified, quantified, and paired‐end sequenced on the Illumina MiSeq platform (Illumina). The raw reads were deposited into the NCBI Sequence Read Archive database (Accession Number: SRP371245), and then analyzed according to the previous study (Zhao et al., 2022).

### 2.10 Multiplex cytokine analysis

Multiplex cytokine analysis is based on multiplex microbead Luminex technology enabling the quantification of multiple analytes in a single assay. Th17 cell and related cytokines can interact with other immune cells and modulate immune responses, which are key contributors to lung injury (Xia et al., 2020). Inflammatory cytokines in the lung tissue were analyzed via a multiplexing magnetic bead‐based antibody detection kit (Milliplex MAP Mouse Th17 Kit, Millipore; Cat: MTH17MAG‐47K) following with the manufacturer's instructions and previous study (Vrselja et al., 2019). The proteins of lung tissues were extracted in ice‐cold modified by using the RIPA kit (Solarbio Life Science). After determining protein concentrations of each sample by BCA protein quantification kit (Beyotime Institute of Biotechnology), 25 μg of protein per sample was loaded onto the multiplex plate for each well approximately. Standard curves for each analyte were generated and the results were analyzed by Bio‐Plex 200 System (Luminex xMAP, BIO‐RAD).

### 2.11 Statistical analysis

Data are expressed as mean ± SEM (standard error of the mean). GraphPad Prism 7.0 was used for all statistical analyses. One‐way ANOVA was used for comparison among the multiple groups. Difference between the pairwise groups was tested for the statistical significance by the student's *t*‐test, and *p* < .05 was considered with the statistical significance.

## 3 RESULTS

### 3.1 POP protects the survival condition, alleviates lung injury, and the immunoinflammatory response

**FIGURE 1:**
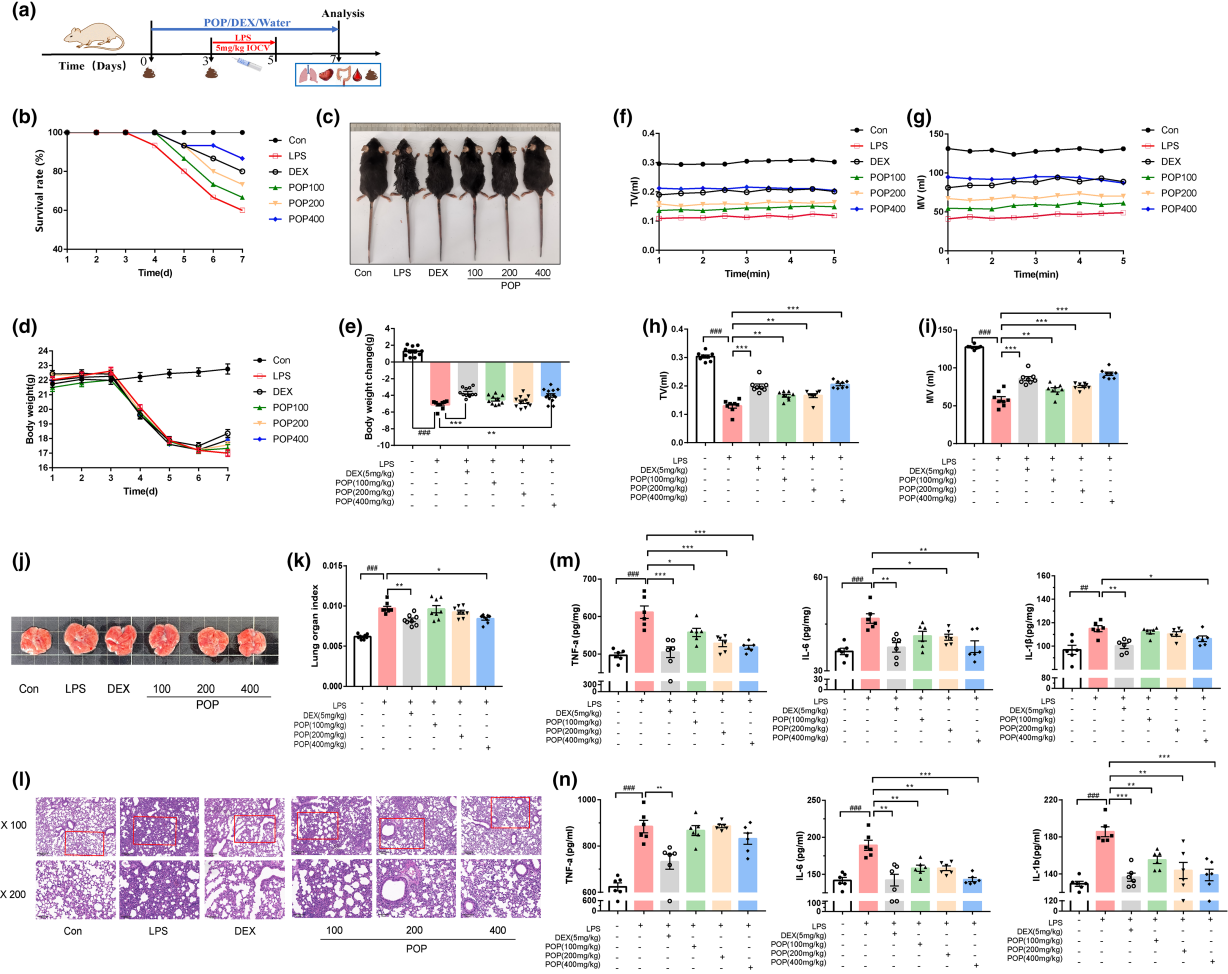
*Polygonatum odoratum* polysaccharide (POP) exerts protective effects on the survival condition and lung injury. (a) The schematic diagram for modeling and administration pipeline in lipopolysaccharide (LPS)‐induced mice; (b) survival rate; (c) representative morphology of mice before euthanasia; (d) body weight of LPS‐induced mice after 7‐day treatment; (e) the change of body weight after 7‐day treatment; Observation of (f) TV and (g) MV in 5 consecutive and steady minutes, and statistical analysis of (h) TV and (i) MV within 10 min; (j) representative picture and (k) the relative weight of lung tissue; (l) representative pictures of hematoxylin–eosin in the lung tissue; The effects of POP on the inflammatory biomarkers (m) in the lung tissue TNF‐α, IL‐6, IL‐1β, and (n) in the serum TNF‐α, IL‐6, and IL‐1β (mean ± SEM, *n* = 6, ^##^ *p* < .01 vs. Con, ^###^ *p* < .001 vs. Con, **p < .01 vs. LPS, ***p < .001 vs. LPS).

The survival rate was 100% in the Con group, whereas that of the LPS group decreased to 60% (*p* < .05) (Figure 1a,b). The DEX and different doses of POP treatment (100, 200, and 400 mg/kg) could significantly improve the survival rate, especially for the mice with high‐dose POP, for which the survival rate could ascend to nearly 90%. Compared to the Con group, the mice in the LPS group exhibited pilomotor fur, mental fatigue, and wet feces. After POP treatment, the spirit, fur, feces, and other characteristics, were ameliorated and generally recovered with dose dependence (vs. LPS) (Figure 1c). Progressive weight gain in the Con group was observed, while the weight decreased continuously from the fourth day until the injection of LPS was stopped. Daily treatment with high dose of POP (400 mg/kg) could significantly reduce the weight loss (*p* < .01, vs. LPS) (Figure 1d,e). As one of the important evaluation indexes for lung injury development, the pulmonary function was examined. The continuous data of mice in the steady breathing state within 5 min were collected, and the average value of tidal volume (TV) and minute ventilation (MV) within 15 min was calculated (Figure 1f–i). Compared with the Con group, the TV and MV values significantly decreased for the LPS group, but significantly increased by the POP treatment in a dose‐dependent manner (400 mg/kg, TV: *p* < .001, MV: *p* < .001). The DEX treatment could also significantly improve the weight loss and the pulmonary function. Thus, the 400 mg/kg‐dose POP intervention not only reduced the mortality rate in mice induced by LPS, but also significantly improved the weight loss and pulmonary function, which thus provided the effectiveness and application potential of POP in the treatment of immuno‐inflammation and lung injury (Devaney et al., 2011; Meng et al., 2018).

Compared with the Con group, pulmonary hemorrhage and edema were noted in the LPS‐treated mice, and treatment with high dose of POP (400 mg/kg) could obviously alleviate this phenomenon (Figure 1j). Moreover, the relative weight of lung tissue was increased in the LPS group compared to the Con group (*p* < .001), while the high dose of POP treatment could decrease it from 0.0097 ± 0.00023 to 0.0084 ± 0.00018 (*p* < .05, vs. LPS) (Figure 1k). Pathological lesions in lung tissue of LPS‐induced inflammatory mice were observed under the light microscopy after H&E staining. Compared with the Con group, the lung staining sections of LPS group exhibited a thickened alveolar septum, marked inflammatory cell infiltration, and increased the proportion of lung parenchyma. After treatment with DEX and high dose of POP, the lung pathological changes were attenuated (vs. LPS), but not observed for the other lower doses of POP groups (Figure 1l). The relative weight of spleen tissue was changed as observed for the lung tissue, after being treated with the high dose of POP (Figure S1a,b). LPS treatment significantly increased hematologic indices of WBC, NE, LY, and MO, which was decreased by the POP treatment in a dose‐dependent manner (Figure S1c–f). Specifically, the high‐dose POP markedly reverted those four hematologic indices mentioned above (WBC, NE, MO: *p* < .001, LY: *p* < .01, vs. LPS). Given that the regulation of pro‐inflammatory cytokines is crucial to prevent excessive inflammation and tissue damage in the lungs (Cheng et al., 2022; Simons et al., 1996), we investigated the effects of POP in regulating the expression of pro‐inflammatory cytokines, including TNF‐α, IL‐1β, and IL‐6, both in the lung and serum samples by ELISA (Figure 1m,n). POP groups could significantly decrease the TNF‐α level in the lung tissue, and the largest decrease was detected in the high‐dose group (*p* < .001, vs. LPS). The high‐dose group showed the downregulated expression of IL‐6 and IL‐1β both in the lung tissue and serum (*p* < .05, vs. LPS).

### 3.2 POP improves intestinal barrier function and gut microbial diversity

**FIGURE 2:**
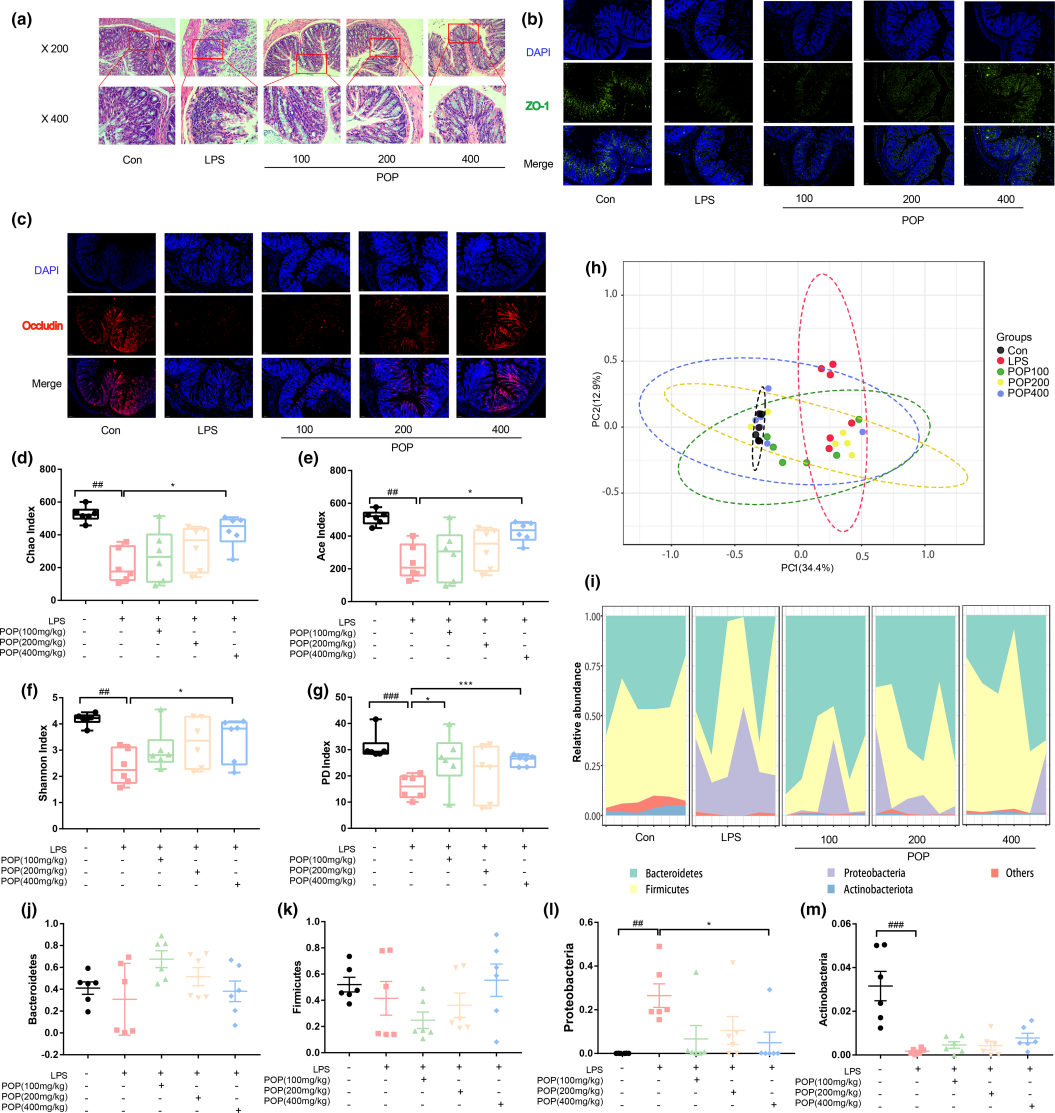
*Polygonatum odoratum* polysaccharide (POP) alleviates the pathological damages of colon tissue and enhances the gut microbial diversity. (a) Representative pictures of hematoxylin–eosin in the colon tissue; representative immunofluorescence photomicrographs for tight junction proteins (b) zonula occludentes (ZO)‐1 (green) and (c) occludin (red) in the colonic epithelium (scale bar equals 50 μm); Alpha‐diversity analysis of different groups including (d) Chao, (e) ACE, (f) Shannon index, and (g) PD index; (h) Beta diversity of Bray–Curtis principal coordinate analysis of different groups; the relative abundance of bacterial communities at (i) total phylum levels and (j) Bacteroidetes, (k) Firmicutes, (l) Proteobacteria, (m) Actinobacteria (mean ± SEM, *n* = 6, ^###^ *p* < .001 vs. Con, ^##^ *p* < .01 vs. Con, *p < .05 vs. lipopolysaccharide [LPS], ***p < .001 vs. LPS).

Literature has reported that, compared with the Con group, LPS injection could induce disordered colon mucosa structure, crypt atrophy, and inflammatory cell infiltration (Chen et al., 2021). In this work, POP intervention ameliorated the colonic injury which was characterized by the disordered colon mucosa structure with crypt atrophy and inflammatory cell reduction (vs. LPS) (Figure 2a). POP treatment also upregulated the expression of intestinal tight junction proteins (e.g., ZO‐1 and occludin) contributing to gut barrier integrity (Figure 2b,c). Particularly, the high‐dose POP treatment displayed remarkable effects on the intestinal barrier function recovery induced by LPS and might be associated with gut microbiota regulation.

LPS could lead to a decreased gut microbial richness (Figure 2d–g). Compared with the Con group, the Chao was significantly decreased in the LPS group (*p* < .01). After high‐dose POP treatment for 7 days, the Chao index was significantly increased (*p* < .05, vs. LPS). The same enhancing trend was detected in the ACE, Shannon, and PD. Additionally, the low‐ and middle‐dose groups could also increase the gut microbial diversity, but no significant difference was observed compared with the LPS group. Principal coordinate analysis (PCoA) indicated the obvious difference between the LPS and Con samples, and the high dose of POP‐treated samples were separated from the LPS group and the other doses‐treated samples (Figure 2h). Compared with the Con group, LPS‐induced mice possessed more Proteobacteria (*p* < .01), but less Firmicutes, Bacteroidetes, and Actinobacteria (*p* < .001) (Figure 2i–m). The high‐dose POP group could decrease the abundance of Proteobacteria in the gut (*p* < .05, vs. LPS).

### 3.3 POP modulates the lung injury‐related bacteria

**FIGURE 3:**
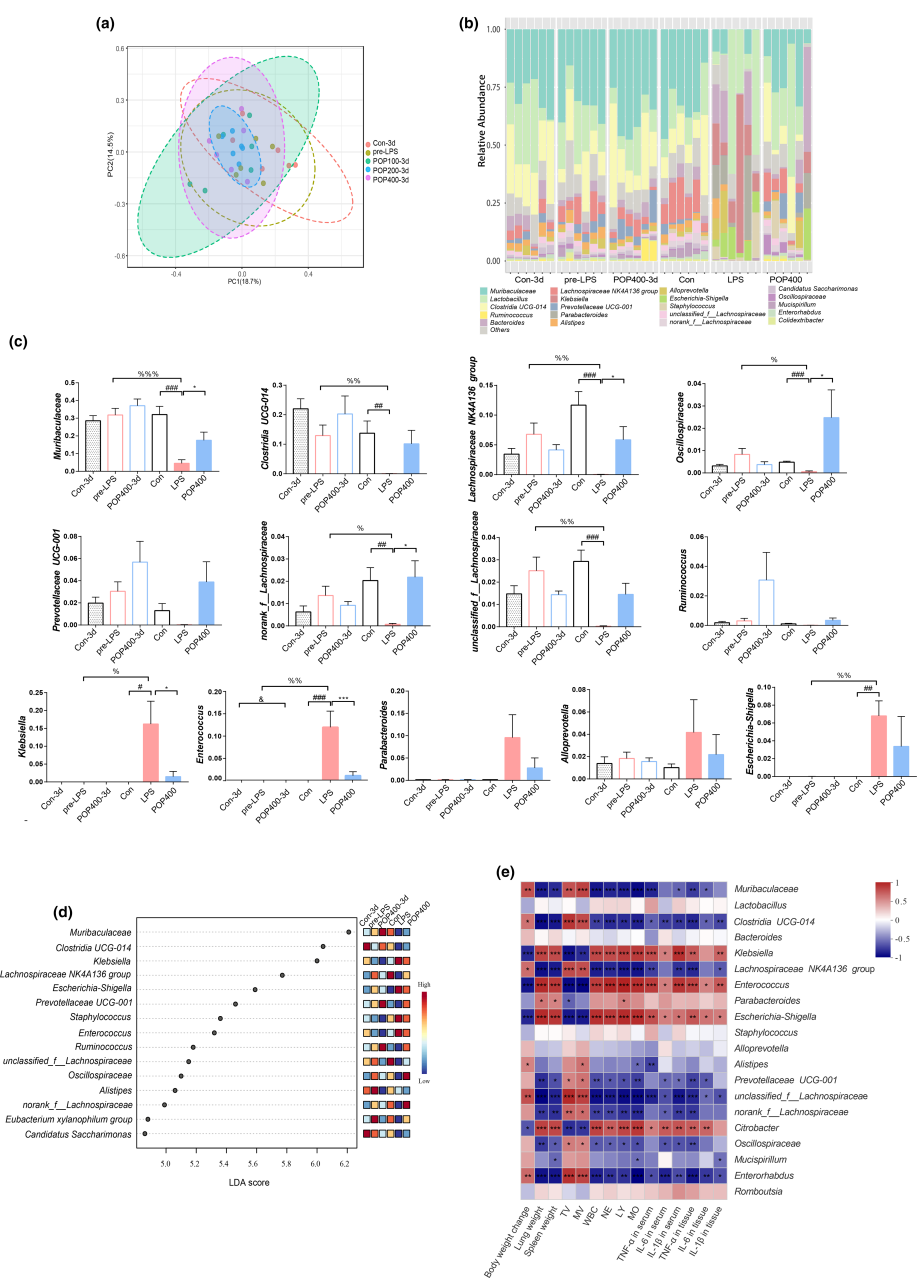
*Polygonatum odoratum* polysaccharide (POP) modulates the immuno‐inflammation and lung injury‐associated gut microbes for both 3‐day and 7‐day treatment. (a) Beta diversity of Bray–Curtis principal coordinate analysis of different groups with POP (Con, 100, 200, and 400 mg/kg) for 3‐day pretreatment; The relative abundance of bacterial communities at (b) total genus levels of Con‐3d, pre‐lipopolysaccharide (LPS), 400‐3d, Con, LPS and 400; (c) statistical analysis of genus whose abundance changed after LPS attacked and was different between high‐dose treatment for 3 and 7 days (mean ± SEM, *n* = 6, ^###^ *p* < .001 vs. Con, ^##^ *p* < .01 vs. Con, ^#^ *p* < .05 vs. Con, ***p < .001 vs. LPS, *p < .05 vs. LPS, ^&^ *p* < .05 vs. Con‐3d, ^%%%^ *p* < .001 vs. pre‐LPS, ^%%^ *p* < .01 vs. pre‐LPS, ^%^ *p* < .05 vs. pre‐LPS); (d) LDA combined with LEfSe analysis; (e) Spearman's correlation analysis between 20 identified bacterial genera and the parameters of lung injury (***p < .001, **p < .01, *p < .05).

The changes of the gut microbiota of mice by LPS modeling and POP administration time were analyzed. Before the LPS injection, the position of different doses of POP‐treated samples could be separated from that of the Con samples in the PCoA (Figure 3a), indicating that the short‐term POP intervention for 3 days could alter the composition of the gut microbiota. Compared with the pre‐LPS group or Con group, the abundance of norank_f_*Muribaculaceae* (*Muribaculaceae*), norank_f_norank_o_*Clostridia* UCG‐014 (*Clostridia* UCG‐014), *Lachnospiraceae* NK4A136 group, norank_f_*Oscillospiraceae* (*Oscillospiraceae*), *Prevotellaceae* UCG‐001, norank_f_*Lachnospiraceae*, unclassified_f_*Lachnospiraceae*, and *Ruminococcus* was decreased; and the abundance of *Klebsiella*, *Enterococcus*, *Parabacteroides*, *Alloprevotella*, and *Escherichia‐Shigella* was increased after LPS modeling, which was corrected after high dose of POP 7‐day treatment (Figure 3b,c). Our results also showed that the abundance of *Muribaculaceae, Prevotellaceae* UCG‐001, and *Ruminococcus* was upregulated after POP treatment for 3 days before LPS attacked, and maintained after LPS modeling, which played important roles in gut microbiota for preventing LPS‐induced injury.

LDA combined with LEfSe was applied to identify the fecal microbial most affected among these groups. *Muribaculaceae, Prevotellaceae* UCG‐001, and *Ruminococcus* were mostly enriched in the POP400‐3d group, and norank_f_*Lachnospiraceae* and *Oscillospiraceae* were mostly enriched in the 400 group with POP for 7 days (Figure 3d). Spearman correlation analysis revealed that bacteria including *Muribaculaceae, Clostridia* UCG‐014, *Lachnospiraceae* NK4A136 group, *Prevotellaceae* UCG‐001, unclassified_f_*Lachnospiraceae*, norank_f_*Lachnospiraceae*, and *Oscillospiraceae* exhibited significantly positive correlation with the body weight change, the pulmonary function (lung weight, TV, MV), and the immunoinflammatory response, whereas, bacteria including *Klebsiella*, *Enterococcus*, and *Escherichia‐Shigella* showed the negative correlation (Figure 3e).

### 3.4 FMT from POP‐fed mice attenuates gut dysbiosis and lung injury

**FIGURE 4:**
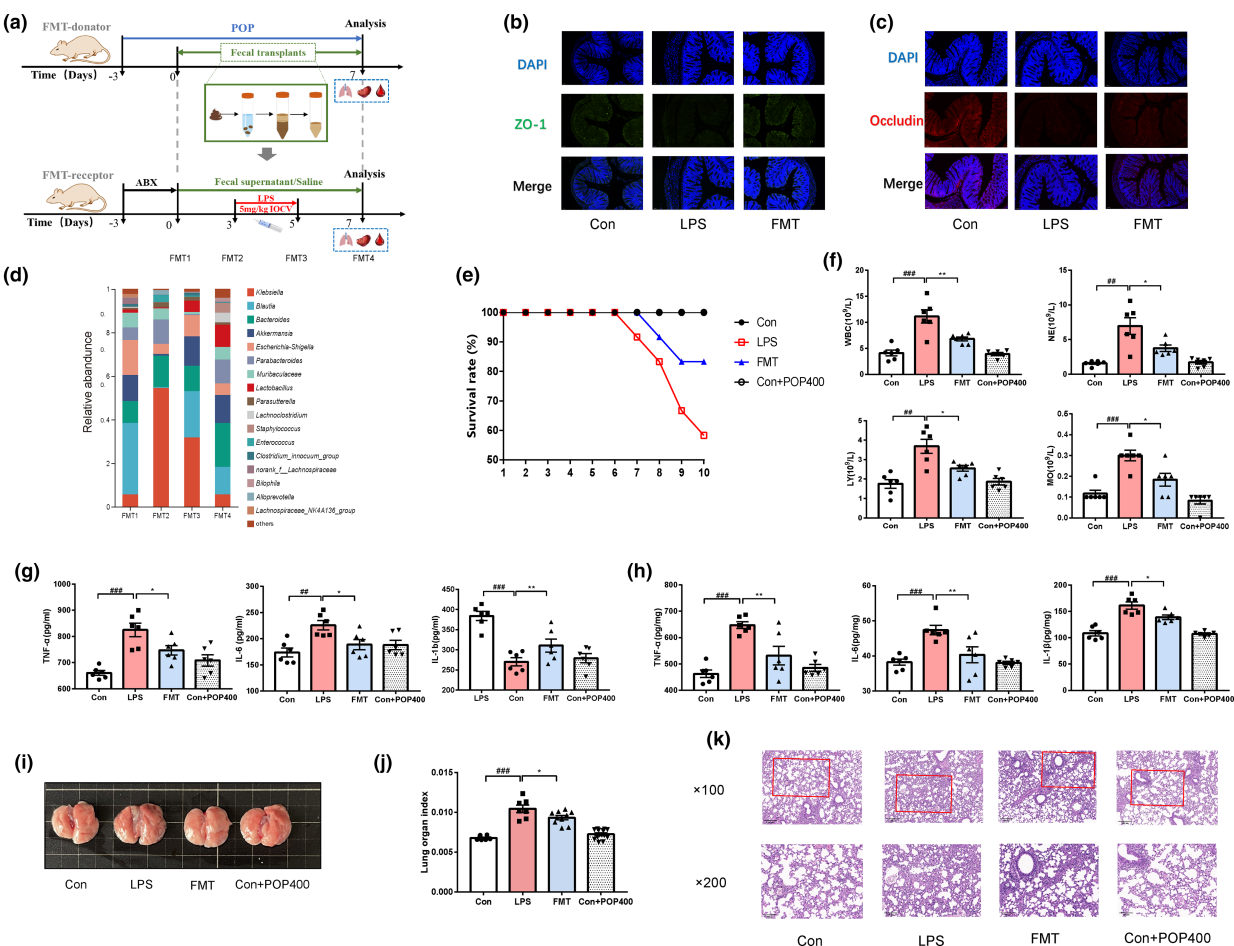
Fecal microbiota transplantation (FMT) improves the immuno‐inflammation and lung injury in lipopolysaccharide (LPS)‐induced mice. (a) study design for the FMT; (b) zonula occludentes (ZO)‐1 (green) and (c) occludin (red) in the colonic epithelium (scale bar equals 50 μm); (d) the relative abundance of bacterial communities at genus levels; (e) survival rate; The effect of *Polygonatum odoratum* polysaccharide (POP) on (f) the blood immune cells analysis WBC, NE, LY, and MO; the inflammatory biomarkers TNF‐α, IL‐6, IL‐1β (g) in the serum, and (h) in the lung tissue; (i) representative picture of lung tissue and (j) the relative weight of lung tissue; (k) representative pictures of hematoxylin–eosin in the lung tissue (mean ± SEM, *n* = 6, ^##^ *p* < .01 vs. Con, ^###^ *p* < .001 vs. Con, *p < .05 vs. LPS, **p < .01 vs. LPS).

Fecal microbiota from POP‐fed mice were transplanted into LPS‐injected recipients (Figure 4a). Notably, the gut barrier protective effects of POP were transferable by fecal transplantation. These observations were accompanied by increased expression of ZO‐1 and occludin (Figure 4b,c). In order to evaluate the effectiveness of FMT, we observed the alteration of gut microbiota during FMT (Figure S2a,b). FMT treatment remodeled the gut microbiome after antibiotic administration and LPS induction, characterized by decreasing the abundance of phylum Proteobacteria, and increasing the abundance of Firmicutes (Figure S2c,d). FMT treatment enriched the abundance of *Lactobacillus*, and reduced the abundance of *Klebsiella* (Figure S2e–f). The abundance of *Lactobacillus, Precotellaceae* UCG‐001, and unclassified_f_*Lachnospiraceae* also had an up‐regulation effect after eating POP in normal donor mice (Figure S2g–h).

Given that FMT ameliorated the gut dysbiosis, we examined whether the beneficial effects of POP may be mediated by the gut microbiota. Compared with the LPS group, the survival rate of FMT group increased from 58.33% to 83.33% (Figure 4e), which was consistent with the previous study (Tang et al., 2021). The body weight of FMT mice was slightly increased, and the pulmonary function was significantly improved (vs. LPS) (Figure S3a–g). Notably, FMT significantly decreased the number of immune cells including WBC, NE, LY, and MO in peripheral blood, compared with the LPS group (Figure 4f). Also, the relative weight of spleen was significantly reduced by FMT (Figure S3h–i, *p* < .01, vs. LPS). In addition, FMT also reduced the level of TNF‐α, IL‐6, and IL‐1β both in serum (Figure 4g) and lung tissue (Figure 4h). These results indicated that the gut microbiota contributed to the anti‐immunoinflammatory effects performed by POP. Recent studies showed that FMT not only improved immunoinflammatory response, but also had certain potential for respiratory disease (Liu et al., 2017). As shown in Figure 4i–k, the relative weight of the lungs was reduced (*p* < .05, vs. LPS), and the pathological damage was reversed, which showed that the alveolar wall thickness was close to Con group, and the inflammatory cell infiltration in the lung space was reduced. FMT treatment also restored the structure of colon and reduced infiltration of inflammatory cells (Figure S3j).

### 3.5 POP and FMT exert the anti‐inflammatory effect by suppressing M1 macrophage in lung

**FIGURE 5:**
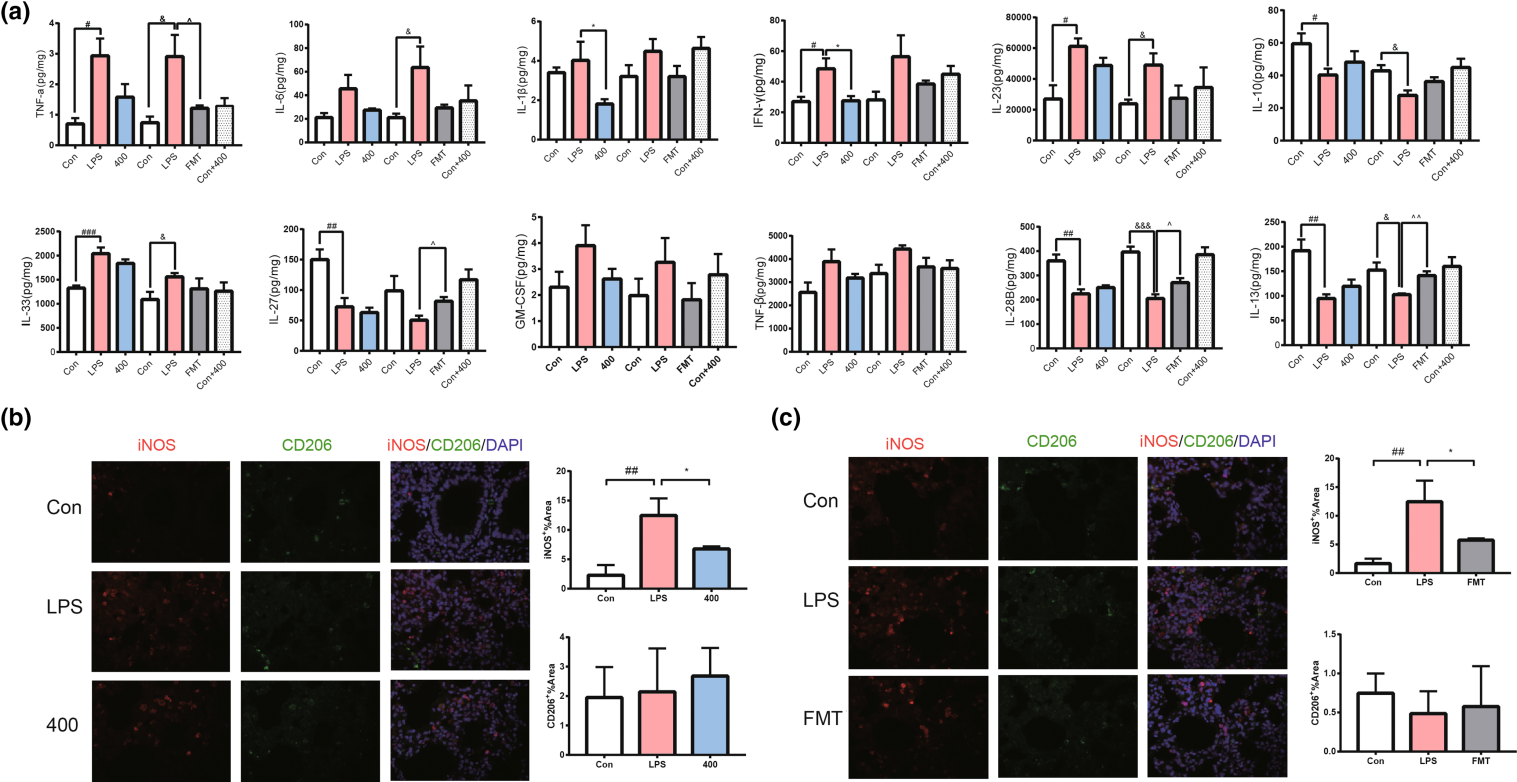
*Polygonatum odoratum* polysaccharide (POP) suppresses the pro‐inflammation in lung. (a) The effects of POP on the multiplex cytokines in the lung tissue (*n* = 5); (b, c) iNOS (red) and CD206 (green) were observed by immunolabeling and fluorescence microscope imaging detection in mice lung tissue (scale bar equals 50 μm) among different groups. The positive area ratio was used to be semiquantitative analysis (*n* = 3) (mean ± SEM, ^###^ *p* < .001 vs. Con, ^##^ *p* < .01 vs. Con, ^#^ *p* < .05 vs. Con, ^&&&^ *p* < .001 vs. Con, ^&^ *p* < .05 vs. Con, *p < .05 vs. lipopolysaccharide [LPS], ^^^^ *p* < .01 vs. LPS, ^^^ *p* < .05 vs. LPS).

The associated molecular mechanisms of POP on immuno‐inflammation and lung injury were investigated. The releases of TNF‐α, IL‐1β, IL‐6, IL‐28B, IL‐13, IFN‐γ, IL‐23, IL‐10, IL‐33, IL‐27, GM‐CSF, TNF‐β, sCD40L, IL‐2, IL‐4, IL‐5, IL‐12, IL‐15, IL‐17A, IL‐25, IL‐17F, IL‐21, IL‐22, IL‐31, and CCL20 after POP and FMT treatment were detected in lung tissue. As shown in Figure 5a, induction with LPS increased the levels of TNF‐α, IL‐1β, IL‐6, IFN‐γ, IL‐23, IL‐10, IL‐33, IL‐27, GM‐CSF, and TNF‐β, and decreased the level of IL‐28B and IL‐13 in lung. In contrast, treatment of POP significantly attenuated the ability of LPS to recover the levels of cytokines. Notably, FMT inhibited the expression of TNF‐α, IL‐1β, IL‐6, and IFN‐γ, promoted the expression of IL‐28B and IL‐13, which demonstrated that the suppression of pro‐inflammatory cytokines was related to the gut microbiota modulation. To explore the effectors underlying POP relieving lung inflammation, we performed immunofluorescence analysis of macrophage activation involved in inflammation (Chen et al., 2020). The results showed that POP and FMT downregulated the iNOS^+^ macrophages, which were presenting the M1 macrophage. But there was no obvious effect on CD206^+^ macrophages (Figure 5b,c). Our results suggested that POP ameliorated lung injury through suppression of the immuno‐inflammation, which was in association with modulating the gut microbiota.

## 4 DISCUSSION

*P. odoratum* have Yin‐nourishing and lung‐moistening functions in TCM, and are often used as functional foods to benefit the health. Polysaccharides isolated from *P. odoratum* can exhibit milder immunomodulatory activity on cell viability and IL‐6 production of RAW 264.7 macrophages, and promote the activity of T LYs in vitro (Zhao et al., 2019). In this study, the therapeutic effects of POP against systemic and pulmonary cytokine release syndrome caused by pathogens such as bacteria or viruses were explored in vivo. Using the LPS‐induced lung inflammatory injury mice model (Lv et al., 2023), the therapeutic potential of POP as a lung protective agent has been confirmed, and its beneficial activity of modulating the associated gut microbiota. Besides, the 400 mg/kg‐dose POP interventions not only reduced the mortality rate from 40% to 10% induced by LPS in mice, but also significantly improved the weight loss, pulmonary function, and pulmonary edema.

Previous studies (Agudelo‐Ochoa et al., 2020; Zuo et al., 2020) have shown that the gut microbiota of patients with lung injury significantly changed compared with normal subjects, which is characterized by enrichment of opportunistic pathogens and depletion of beneficial commensals. LPS can disrupt the integrity of the gut barrier, leading to increased intestinal permeability (Yoseph et al., 2016), which allows the translocation of bacterial components, including LPS itself, into the bloodstream, resulting in amplification of the systemic inflammatory response and contribution to exacerbation of lung injury (Dickson, 2016). The immune activation and inflammation can disrupt the delicate balance of the gut microbiota, resulting in changes in its diversity and abundance. In turn, gut microbiota dysbiosis can contribute to the translocation of microbial components from the gut lumen into systemic circulation, which is a potential factor of inflammation and immune‐related pathology (Bai et al., 2022; Pickard et al., 2017), and further affect pulmonary immunity through a vital cross‐talk between gut and lung, as gut–lung axis. Alterations in the gut microbial species and metabolites have been linked to changes in immune responses and inflammation as well as the lung injury development. As a signature feature of gut dysbiosis, the abundance of Proteobacteria was significantly decreased in the high‐dose group (*p* < .05, vs. LPS), suggesting a closely link with the therapeutic effects of POP (Shin et al., 2015). The enhancement of the proportion of *Lactobacillus* (Hossain et al., 2022), *Muribaculaceae* (Tian et al., 2021), and *Prevotellaceae* UCG‐001 (Zhu et al., 2021), the SCFA‐producing bacteria, can mitigate colonic barrier dysfunction and inflammation and inhibit the endotoxin production. Additionally, the reduction of harmful bacteria including *Escherichia‐shigella* and *Klebsiella*, which were the typical LPS producing, gram‐negative bacteria, had been confirmed to ameliorate the lung injury (Sun et al., 2020). Notably, *Klebsiella* could disrupt the gut barrier (Nakamoto et al., 2019), which not only trigger a systemic or local inflammatory response that further altering the overall immune homeostasis of the host to exacerbate the occurrence of inflammatory lung injury (Belkaid & Hand, 2014), but also lead to a risk factor for pneumonia‐induced lung injury (Liu et al., 2018). Tang et al. (2021) stablished a gut flora disorder model with administration antibiotics in LPS‐induced acute lung injury mice, and found that FMT improved the imbalance and diversity of the gut flora‐induced and ‐antagonized LPS. The gut microbiota was modulated by a short‐term (3‐day) POP pre‐intervention and the trend was sustained of *Lactobacillus* in the donor mice, which played important roles in gut microbiota for prevention in LPS‐induced injury. *Lactobacillus*, a genus of beneficial bacteria, can inhibit the growth and activity of *Klebsiella* (Tang et al., 2022). As a potential prebiotic‐like polysaccharide, POP might promote the growth of *Lactobacillus* to inhibit the growth and activity of *Klebsiella*. Our results indicated that the therapeutic mechanism of POP that are not directly absorbed was associated with the remodeling the gut microbiota.

Moreover, the observed gut microbiota composition shift might be a direct response to the POP's administration, so as to reach the anti‐immune inflammatory states via the innate immune signaling pathway, rather than a direct involvement in disease severity by POP (Ji et al., 2014). Our results suggested that POP alleviated LPS‐induced immuno‐inflammation and lung injury in association with modulating gut microbiota, which may be related to the inhibition of inflammation, protection of intestinal barrier, and regulation of mucosal immunity through a vital cross‐talk between gut and lung, as the gut–lung axis (Yang & Cong, 2021). The results of TNF‐α, IL‐1β, and IL‐6 were consistent with the previous results during the lung inflammation development. IL‐28B and IL‐13 may also modulate the lung inflammation through suppressing the generation of pro‐inflammatory molecules (Yan et al., 2017). While using the multiplex Luminex panel to evaluate the cytokine expression was more sensitive and advanced, allowing the detection of more factor changes (Johnson et al., 2019). It has been reported that the TLR4 signaling pathway played an important role in the development of pro‐inflammatory cytokine expression and M1 macrophage activation (Zhang et al., 2022). TLR4 was a sensor mediating the crosstalk between the intestinal commensal microbiome and host immunity, and the gut commensal present significant anti‐inflammation effects during microbial respiratory infections by inhibiting TLR4 and its signal transduction (Wu et al., 2022). LPS‐induced inflammation was involved in the activation of MAPKs, NF‐κB, and JAK signal pathway (Lai et al., 2017) thereby resulting in the overproduction of cytokines, such as TNF‐α, IL‐1β, and IL‐6 (Dang & Marsland, 2019). Our results suggested that POP ameliorated the lung injury through suppression of pro‐inflammatory M1 macrophages, which was in association with modulating the gut microbiota. Polysaccharides can modulate the gut microbiota composition and production of metabolites, such as SCFAs, leading to anti‐inflammatory effects. More work is needed to understand the mechanisms, and context, for how gut microbes or metabolites, selectively prime myeloid precursors in the gut–lung axis upon inflammation and if this also holds true during homeostasis (Dang & Marsland, 2019; Nan et al., 2023). Additional studies on the underlying mechanisms could give valuable insight on how to modulate the immune system at one of its most fundamental levels for therapeutic purposes.

In conclusion, POP could significantly ameliorate the immuno‐inflammation and lung injury by modulating the gut microbiota, protecting the intestinal barrier, inhibiting the M1 macrophages in LPS‐induced inflammatory injury mice. Our findings elucidated the biological basis of POP to improve lung inflammatory injury from the perspective of regulating gut microbiota, and provided new ideas to develop the nutritional prevention and treatment strategy of lung protection based on the gut. These results favor that POP may be used as a prebiotic agent for targeting the gut microbiota against the inflammation‐related diseases.

## AUTHOR CONTRIBUTIONS

**Jia‐rui Liu:** Data curation (lead); writing – original draft (lead). **Bo‐xue Chen:** Data curation (supporting); writing – original draft (supporting). **Mei‐ting Jiang:** Investigation (equal); methodology (equal). **Tian‐yi Cui:** Investigation (equal); methodology (equal). **Bin Lv:** Investigation (equal); methodology (equal). **Zhi‐fei Fu:** Investigation (equal); methodology (equal). **Xue Li:** Investigation (equal); methodology (equal). **Yao‐dong Du:** Visualization (equal). **Jin‐he Guo:** Visualization (equal). **Xin‐qin Zhong:** Visualization (equal). **Ya‐dan Zou:** Visualization (equal). **Xin Zhao:** Funding acquisition (equal); supervision (equal); writing – review and editing (equal). **Wen‐zhi Yang:** Funding acquisition (equal); supervision (equal); writing – review and editing (equal). **Xiu‐mei Gao:** Funding acquisition (equal); supervision (equal).

## FUNDING INFORMATION

This work was supported by the National Key R&D Program of China (2022YFC3500300; 2022YFC3500305), the Innovation Team and Talents Cultivation Program of National Administration of Traditional Chinese Medicine (ZYYCXTD‐C‐202009), National Natural Science Foundation of China (81872996), and Tianjin Municipal Education Commission Research Project (2022ZD039).

## CONFLICT OF INTEREST STATEMENT

The authors declare no conflict of interest.

## Supporting information


Data S1.

## Data Availability

The sequence datasets presented in this study can be found in online repositories. The names of the repository and accession number can be found at: https://www.ncbi.nlm.nih.gov/, SRP371245. The other data that support the findings of this study are available on request from the corresponding author.
