# Supplementary material for: Polygonatum odoratum polysaccharide attenuates lipopolysaccharide‐induced lung injury in mice by regulating gut microbiota
Source: Food Sci Nutr. 2023 Aug 15;11(11):6974–86. doi: 10.1002/fsn3.3622 (PMC10630852; doi:10.1002/fsn3.3622)
Supplement: Supplementary file 1 — Data S1. [file FSN3-11-6974-s001.doc]

**<** ***Food Science and Nutrition* >**

Supporting Information

***Polygonatum odoratum* polysaccharide attenuates lipopolysaccharide-induced lung injury in mice by regulating gut microbiota**

Jia-rui Liu ^a,b,#^, Bo-xue Chen ^b,#^, Mei-ting Jiang ^b^, Tian-yi Cui ^a,b^, Bin Lv ^a,b^, Zhi-fei Fu ^a,b^, Xue Li ^b^, Yao-dong Du ^a,b^, Jin-he Guo ^a,b^, Xin-qin Zhong ^a,b^, Ya-dan Zou ^b^, Xin Zhao ^a,b*^, Wen-zhi Yang ^a,b,*^, Xiu-mei Gao ^a,b,*^

^a^ Ministry of Education Key Laboratory of Pharmacology of Traditional Chinese Medical Formulae, Tianjin University of Traditional Chinese Medicine, 10 Poyanghu Road, Jinghai, Tianjin 301617, China

^b^ State Key Laboratory of Component-based Chinese Medicine, Tianjin University of Traditional Chinese Medicine, 10 Poyanghu Road, Jinghai, Tianjin 301617, China

^*^ Corresponding author

**Xin Zhao**: Tel: 86-22-59596164; Fax: 86-22-59596164; E-mail: x.zhao26@tjutcm.edu.cn (Xin Zhao), wzyang0504@tjutcm.edu.cn (Wen-zhi Yang), gaoxiumei@tjutcm.edu.cn (Xiu-mei Gao)

^#^ Authors contributed equally to this work.

**Content**

**Figure S1** POP inhibits the immuno-inflammatory response. (A) Representative picture of and (B) the relative weight of spleen tissue; The effect of POP on the blood immune cells (C-F) WBC, NE, LY, and MO (Mean±SEM, n=6, ##*p < 0.01* versus Con, ###*p < 0.001* versus Con, **p < 0.05* versus LPS, ***p < 0.01* versus LPS, ****p < 0.001* versus LPS).

**Figure S2** The microbial regulated effects of FMT on LPS-induced mice. (A) Timeline of FMT receptor; (B) Beta diversity of bray_curtis PCoA of different time pots during FMT; (C-D) Statistical analysis of phylum after FMT treatment; (E-F) Statistical analysis of genus after FMT treatment; (G) Timeline of POP treatment as FMT donor; (H) The relative abundance of bacterial communities at genus levels after POP treatment (Mean±SEM, *n*=6, ###*p < 0.001* versus FMT1, ##*p < 0.01* versus FMT1, &&&*p < 0.001* versus FMT2, &&*p < 0.01* versus FMT2, &*p < 0.05* versus FMT2, ***p < 0.01* versus FMT3, **p < 0.05* versus FMT3).

**Figure S3** The therapeutic effects of FMT on LPS-induced mice. (A) Representative morphology of mice before euthanasia; (B) Body weight of LPS-induced mice after 7-day treatment; (C) The change of body weight after 7-day treatment; Observation of (D) TV and (E) MV in 5 consecutive and steady minutes, and statistical analysis of (F) TV and (G) MV within 10 minutes; (H) Representative picture of spleen tissue; (I) the relative weight of spleen tissue; (J) Representative pictures of H&E in the colon tissue (Mean ± SEM, Con group *n*=6, the other groups n=12, ###*p < 0.001* versus Con, **p < 0.05* versus LPS, ***p < 0.01* versus LPS).


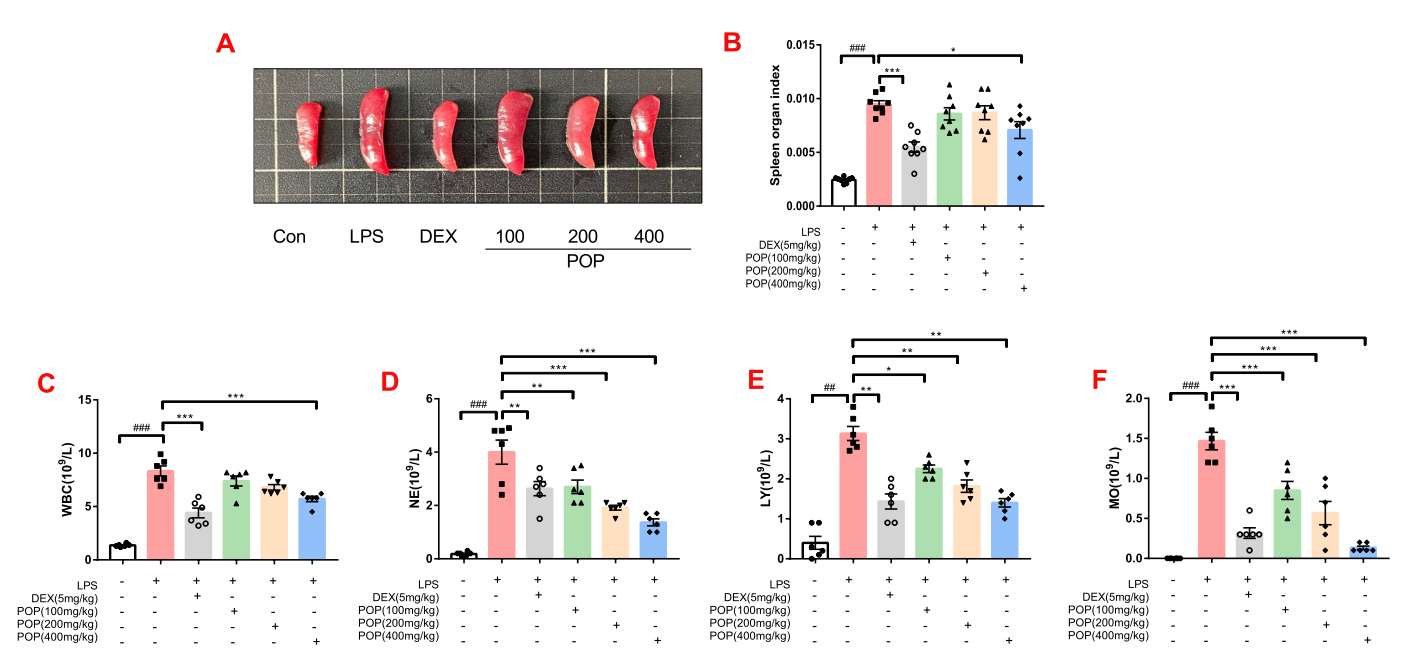


**Figure S1** POP inhibits the immuno-inflammatory response. (A) Representative picture of and (B) the relative weight of spleen tissue; The effect of POP on the blood immune cells (C-F) WBC, NE, LY, and MO (Mean±SEM, n=6, ##*p < 0.01* versus Con, ###*p < 0.001* versus Con, **p < 0.05* versus LPS, ***p < 0.01* versus LPS, ****p < 0.001* versus LPS).


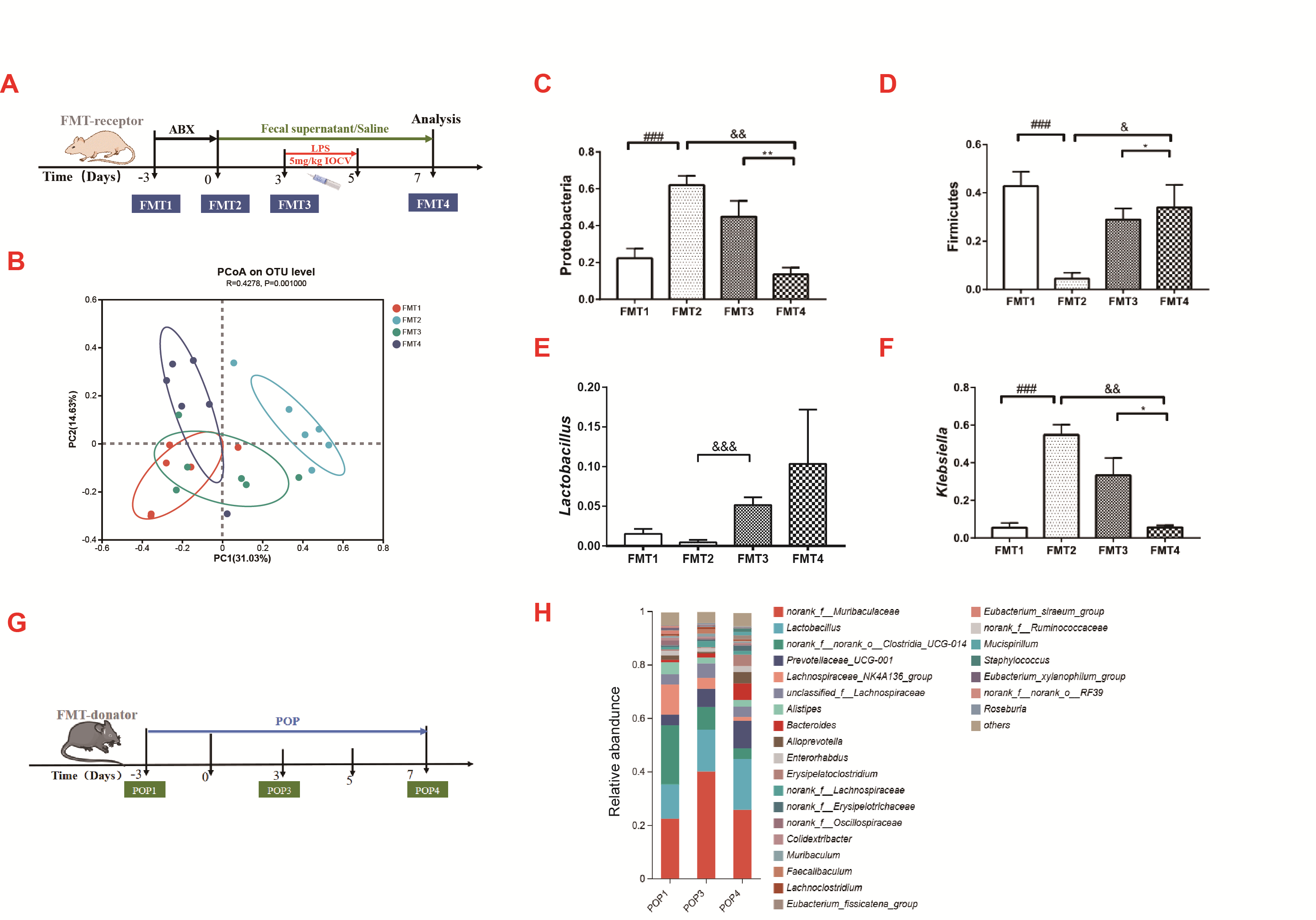


**Figure S2** The microbial regulated effects of FMT on LPS-induced mice. (A) Timeline of FMT receptor; (B) Beta diversity of bray_curtis PCoA of different time pots during FMT; (C-D) Statistical analysis of phylum after FMT treatment; (E-F) Statistical analysis of genus after FMT treatment; (G) Timeline of POP treatment as FMT donor; (H) The relative abundance of bacterial communities at genus levels after POP treatment (Mean±SEM, *n*=6, ###*p < 0.001* versus FMT1, ##*p < 0.01* versus FMT1, &&&*p < 0.001* versus FMT2, &&*p < 0.01* versus FMT2, &*p < 0.05* versus FMT2, ***p < 0.01* versus FMT3, **p < 0.05* versus FMT3).


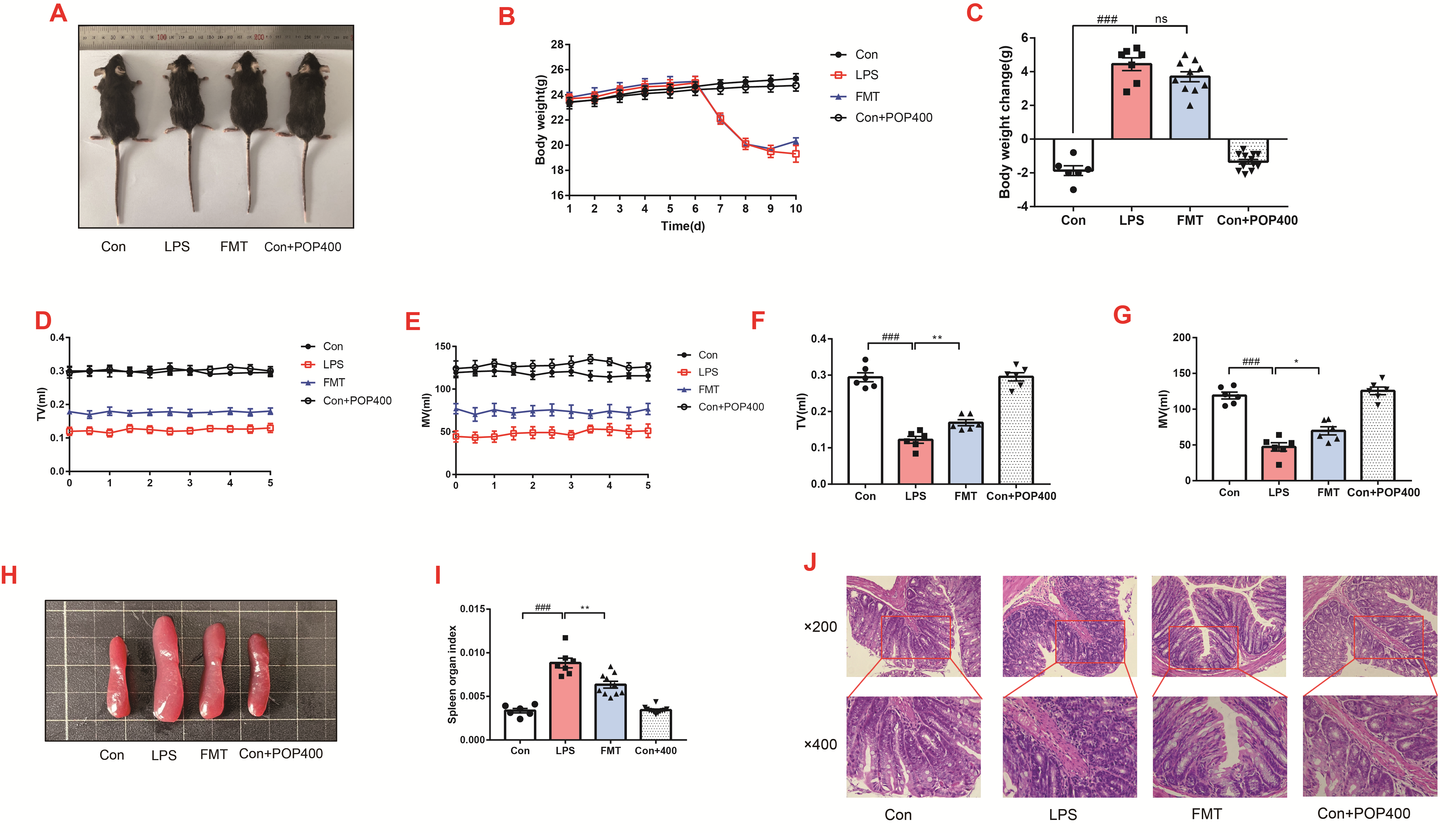


**Figure S3** The therapeutic effects of FMT on LPS-induced mice. (A) Representative morphology of mice before euthanasia; (B) Body weight of LPS-induced mice after 7-day treatment; (C) The change of body weight after 7-day treatment; Observation of (D) TV and (E) MV in 5 consecutive and steady minutes, and statistical analysis of (F) TV and (G) MV within 10 minutes; (H) Representative picture of spleen tissue; (I) the relative weight of spleen tissue; (J) Representative pictures of H&E in the colon tissue (Mean ± SEM, Con group *n*=6, the other groups n=12, ###*p < 0.001* versus Con, **p < 0.05* versus LPS, ***p < 0.01* versus LPS).

**Table S1.** Total sugar contents of the POP using standard curve of glucose by UV spectrophotometer.

| **Analyte** | **Total Sugar (%)** | **Regression equation** | ***R*^2^** | **Concentration**  **(µg/mL)** |
| --- | --- | --- | --- | --- |
| Standards | - | *y*=0.0085X-0.0134 | 0.9996 | 4,8,16,31,62,125,250 |
| POP | 66.36 | - | - | 125 |
